# Supplementary material for: Genome-Wide Identification and Expression Analysis of MAPK Gene Family in Lettuce (Lactuca sativa L.) and Functional Analysis of LsMAPK4 in High- Temperature-Induced Bolting
Source: Int J Mol Sci. 2022 Sep 22;23(19):11129. doi: 10.3390/ijms231911129 (PMC9569992; doi:10.3390/ijms231911129)
Supplement: Supplementary file 1 [file ijms-23-11129-s001.zip › Supplement Table and Figure Legends.pdf]

Table S1. Primers for qRT–PCR

Figure S1. Sequence alignment between LsMAPK4 and LsMAPK4 *. (**A**) Gene sequence alignment. (**B**) Open Reading Frame sequence alignment.

Figure S2. Multiple sequence alignment of LsMAPKs
